# Patterns of Spontaneous Local Network Activity in Developing Cerebral Cortex: Relationship to Adult Cognitive Function

**DOI:** 10.1371/journal.pone.0131259

**Published:** 2015-06-22

**Authors:** Alejandro Peinado, Charles K. Abrams

**Affiliations:** Department of Neurology and Department of Physiology and Pharmacology, State University of New York, Downstate Medical Center, Brooklyn, New York, United States of America; University Paris 6, FRANCE

## Abstract

Detecting neurodevelopμental disorders of cognition at the earliest possible stages could assist in understanding them mechanistically and ultimately in treating them. Finding early physiological predictors that could be visualized with functional neuroimaging would represent an important advance in this regard. We hypothesized that one potential source of physiological predictors is the spontaneous local network activity prominent during specific periods in development. To test this we used calcium imaging in brain slices and analyzed variations in the frequency and intensity of this early activity in one area, the entorhinal cortex (EC), in order to correlate early activity with level of cognitive function later in life. We focused on EC because of its known role in different types of cognitive processes and because it is an area where spontaneous activity is prominent during early postnatal development in rodent models of cortical development. Using rat strains (Long-Evans, Wistar, Sprague-Dawley and Brattleboro) known to differ in cognitive performance in adulthood we asked whether neonatal animals exhibit corresponding strain-related differences in EC spontaneous activity. Our results show significant differences in this activity between strains: compared to a high cognitive-performing strain, we consistently found an increase in frequency and decrease in intensity in neonates from three lower performing strains. Activity was most different in one strain considered a model of schizophrenia-like psychopathology. While we cannot necessarily infer a causal relationship between early activity and adult cognition our findings suggest that the pattern of spontaneous activity in development could be an early predictor of a developmental trajectory advancing toward sub-optimal cognitive performance in adulthood. Our results further suggest that the strength of dopaminergic signaling, by setting the balance between excitation and inhibition, is a potential underlying mechanism that could explain the observed differences in early spontaneous activity patterns.

## Introduction

An important goal in the neuroscience of mental illness is the early detection of neurodevelopmental disorders prior to the onset of the disabling manifestations of cognitive dysfunction, when intervention may be more effective [1]. In disorders of cognition, such as schizophrenia, where an early developmental etiology is suspected [2], subtle physiological clues may be present very early on. Where and how to look for these early physiological indicators, or “biomarkers”, that precede cognitive dysfunction has not been obvious. In the last 10 to 15 years it has become apparent that in all mammals, including humans, much of the activity occurring in the brain is activity generated internally, independently of sensation and unrelated to voluntary tasks [3]. This is particularly so during early development [4,5,6,7,8,9,10,11,12,13,14], where the spatiotemporal structure of this “spontaneous” activity, often generated independently by local networks, is highly organized and can generate distinctive macroscopic signatures amenable to visualization through different types of functional neuroimaging. We hypothesized that this activity might contain useful information which could both identify early deficits in the basic functional properties of local networks and serve as a predictor of future deficits in the functions associated with those networks.

The periodic pattern of spontaneous coordinated firing in developing networks triggers brief increases in neuronal calcium that are not only a useful proxy for monitoring the electrical activity of these networks but are, just as importantly, a process that can profoundly and sometimes irreversibly influence circuit development through its effect on gene expression and synapse formation [15,16,17,18]. Particularly during developmental “critical periods”, which are periods of heightened circuit plasticity [19], abnormal patterns of activity and calcium influx in neurons can, over time, become codified into dysfunctional local network properties. It is these sub-optimal network properties established early in life that can limit the future functional potential of the affected circuits, as was first shown by Hubel and Wiesel in the visual system [20]. To the extent that this also occurs in local networks associated with cognition it follows that cognitive dysfunction in adulthood is likely to have been preceded by some sub-optimal pattern of activity at some critical time during the early formative stages of these networks. We wondered whether evidence of this could be gleaned in the patterns of spontaneous activity of neonates from rodent strains previously characterized as cognitive underperformers as adults.

Our study, applying low-magnification calcium imaging to brain slices, provides a quantitative characterization of the spatiotemporal structure of spontaneous local network activity in the developing entorhinal cortex (EC) in different rat strains. The results show that there are significant strain differences in the pattern of this activity and that these differences correlate with known differences in cognitive performance between the respective strains. Briefly, we find that strains known for low performance in adulthood exhibit a pattern of EC spontaneous network activity during development that is significantly different from that of the high performing strain. In some of the measured parameters the activity is seen to be significantly weaker in the low-performing strains. We also show that GABA-A-mediated inhibition and D1-mediated dopaminergic modulation are mechanisms likely to underlie the strain-related differences in spontaneous activity.

## Results

### Comparison of three behaviorally well-characterized strains: Long Evans, Wistar and Sprague Dawley

In the first part of our study we compared neonatal activity patterns in one “smart” strain, Long Evans (LE), and two strains, Wistar (W) and Sprague Dawley (SD), that consistently attain significantly lower scores on tests of cognitive performance [21,22,23]. Animals ranged in age between postnatal day (PND) 4 and PND 9. Brain slices containing the hippocampal formation were cut in the horizontal plane at the level of the ventral hippocampus and stained with calcium-sensitive fluorescent dye Fura-2 AM (see Methods). A low magnification (1.75X) was chosen such that the macroscopic pattern of changes in neuronal [Ca^2+^]_in_ could be monitored throughout the entire EC. The field of view could only encompass the EC in one hemisphere. Pixel binning (8x8) and relatively long exposure times (250 msec) were used in order to increase signal-to-noise under relatively low illumination intensity. Fura-2 changes in individual cells could not be resolved with these settings. Images were acquired continuously at 4 frames per second for periods of up to 20 minutes. From the raw data we extracted five parameters to characterize the intensity and temporal structure of EC spontaneous activity in each strain: 1) Frequency of activation, 2) Maximal % rate of rise of EC activation, 3) Variability of activation, 4) % of pixels active above a threshold level, and 5) Relative activation of lateral vs. medial EC. All data are expressed as mean ± S.E.M.

**Fig 1:**
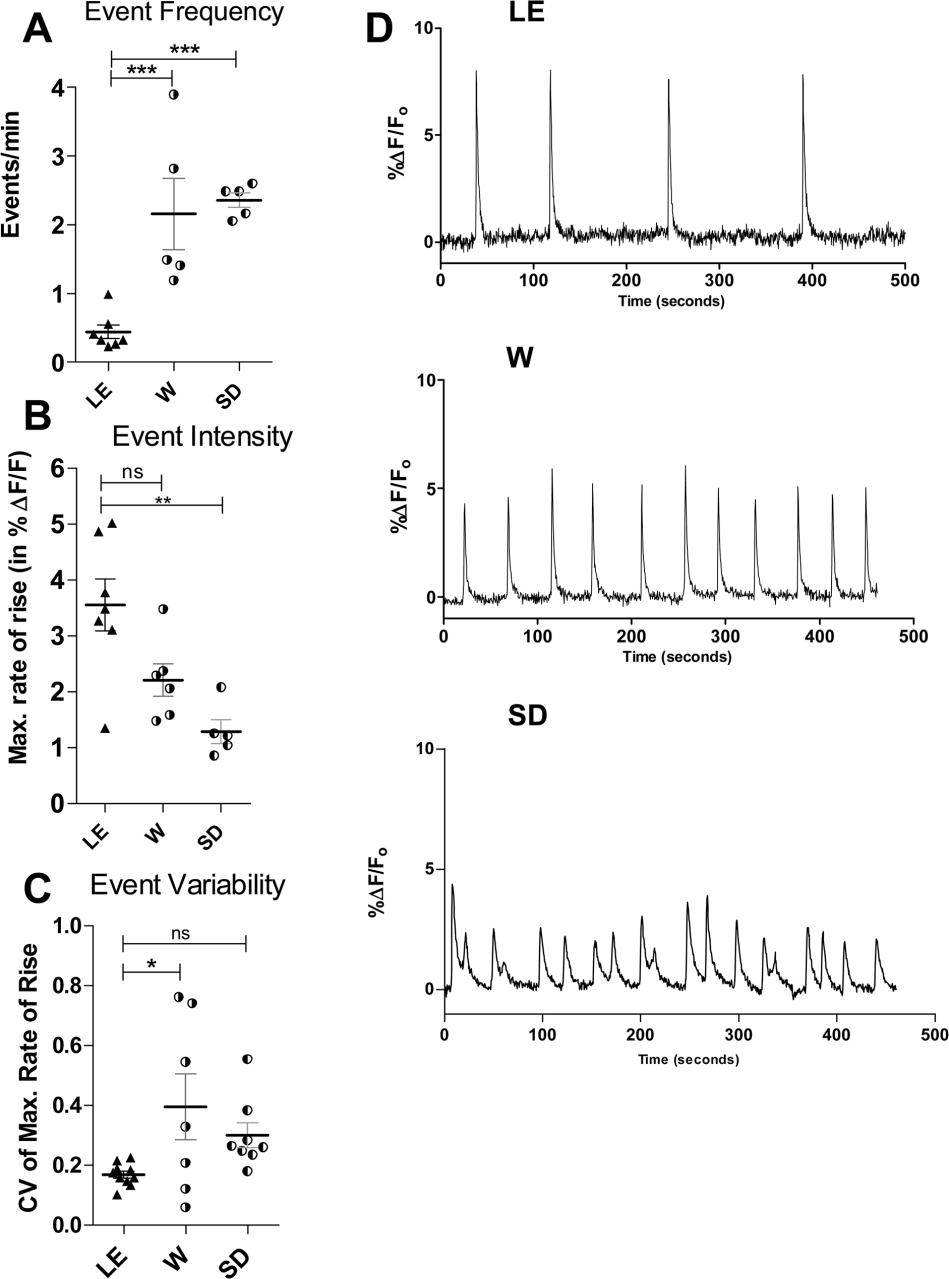
Temporal dynamics of EC spontaneous activity reveal differences in spontaneous network activity between strains. A significantly higher frequency (**A**) of spontaneous events is apparent in W and SD slices relative to LE. In contrast, the intensity of activation (**B**) as measured by the maximal rate of change in the calcium signal at the onset of each event is significantly reduced in both low performing strains. The coefficient of variation (CV) in the activation intensity of both lower performing strains was significantly increased (**C**), demonstrating a greater event-to-event variability in the extent of network recruitment. In all cases data are expressed as mean ± SEM. Event variability is based on CVs computed using all events recorded during each calcium imaging sequence. * p<0.05, ** p<0.01, *** p<0.001, one-way ANOVA with Bonferroni’s post hoc test (data from: LE = 10 slices (7 pups); W = 6 slices (5 pups); SD = 6 slices (5 pups)). **D** shows representative traces of the calcium-dependent fluorescence over time in EC for each of the three strains.

First, we determined the average number of spontaneous events per minute for each strain (Fig 1A). This parameter is of interest as it has been shown that the frequency of spontaneous activity during development can affect connectivity patterns [24] as well as gene expression, including that of dopamine- and GABA-related genes [25]. We found that the frequency of events per minute in LE (0.44± 0.10) is several fold lower than that in both W (2.15 ± 0.52) and SD (2.36 ± 0.10). By one-way ANOVA LE event frequency was significantly lower than that of SD and W (p < 0.001) whereas W and SD frequencies did not differ significantly from each other.

In contrast to the event frequency, the intensity of activation, defined as the maximal rate of change in the calcium signal at the start of each event and obtained by taking the derivative of the calcium signal (Fig 1B), was very significantly higher in LE, averaging approximately twice the rate recorded in W or SD. The magnitude of this measure reflects the proportion of active cells sampled at each pixel as well as the timing and magnitude of the calcium signal in every active cell. As shown in Fig 1B, the activation intensity in LE slices averaged 3.55 (± 0.46) points (in units of %∆F/F_o_), compared to 1.28 (± 0.21) for SD and 2.21 (± 0.29) for W. One-way ANOVA showed LE activation strength to be significantly different from that of SD (p < 0.01) but not W. Wistar activation strength was intermediate and did not differ statistically from that of SD or LE. It should be noted that the regions of interest (ROI) from which these measurements were derived were deliberately selected to obtain a maximal value for each slice (i.e. when activation intensity was not uniform throughout EC a region exhibiting the strongest activation was identified and used). Therefore, this parameter overestimates the overall intensity of activation throughout EC in SD and W slices, and needs to be considered in conjunction with the fourth parameter, described below.

The third parameter we quantified was the variability of activation present in the events occurring within each 10- or 20-minute recording. For this we computed the coefficient of variation (CV = Standard Deviation/Mean) for the maximal rate of rise from all events in each recording sequence. This provides a measure of how consistently the cells within the selected ROI are being excited and are participating from one event to the next. We found that consistency was higher (lower CV) in LE than in either W or SD slices. However, only LE and W slices showed a significant difference (one-way ANOVA; p < 0.01) (Fig 1C).

**Fig 2:**
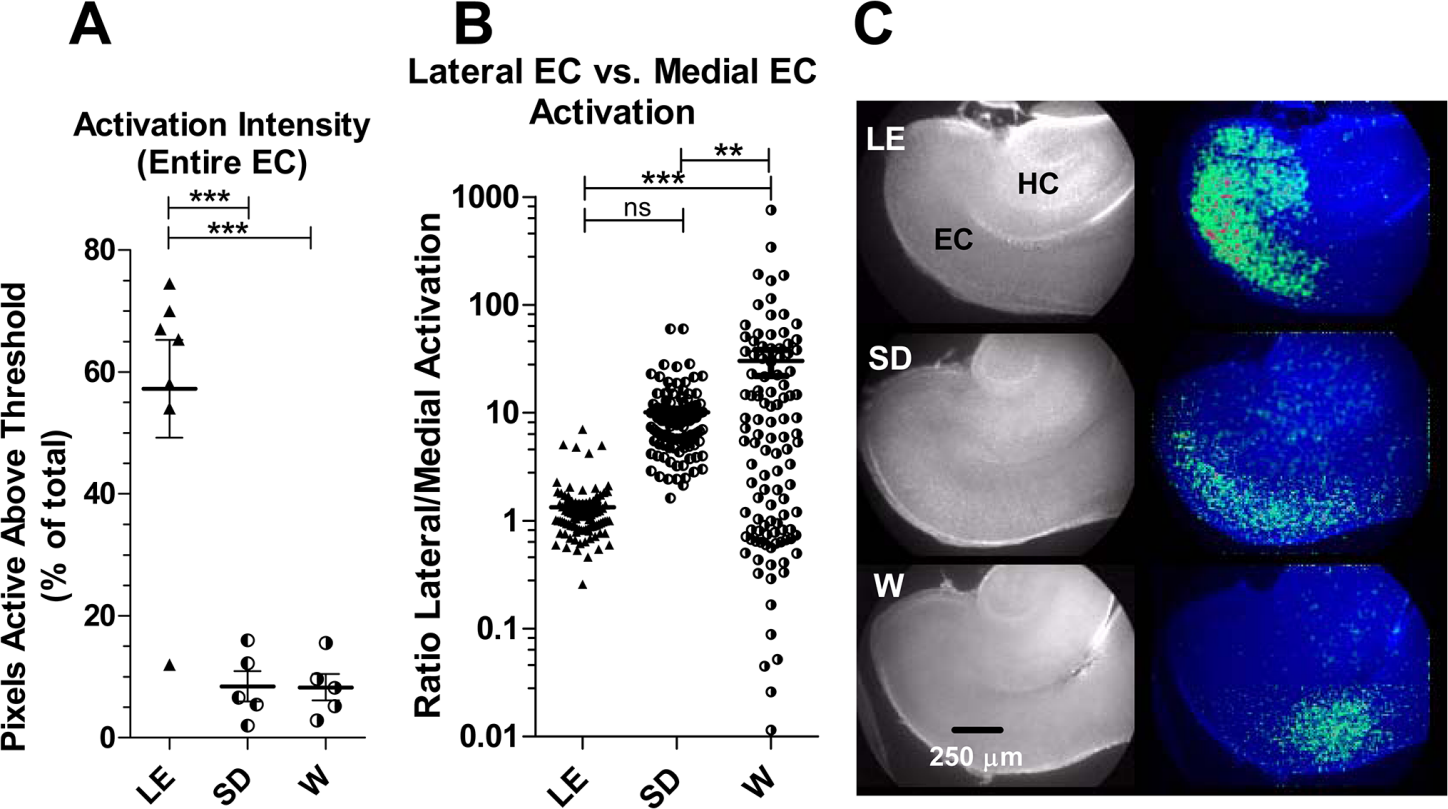
Spatial mapping of EC spontaneous activity reveals reduced activation in W and SD vs. LE. A significant decrease in the overall intensity (**A**) of spontaneous events is apparent in W and SD slices. The intensity of EC activation was calculated as the percentage of total EC pixels active above a threshold value during each coordinated event. **B** shows the localization of activity within EC in terms of the relative activation of lateral vs. medial pixels. Activation exhibits a relatively uniform distribution over all EC in the LE strain. In both W and SD strains activation is higher in the more lateral EC relative to more media ECl. **, p< 0.01; ***, p< 0.001. one-way ANOVA with Bonferroni’s post hoc test (n’s: LE = 10 slices (7 pups); W = 6 slices (5 pups); SD = 6 slices (5 pups)). **C** shows representative composite images illustrating the activated pixels (greens and reds in pseudocolor overlay shown on right) in a slice from each strain. W slice illustrates an extreme example of activation with a lateral bias. HC, hippocampus; EC, entorhinal cortex; In every slice medial is up, posterior is left.

The fourth parameter examined was the fraction of pixels in EC activated above a predetermined threshold during each spontaneous event. This is a more global measure of activation intensity in EC and complements the more local measure of intensity reported above (this measure included pixels comprising all layers, spanning all EC from the most medial end to the constriction at the rhinal sulcus). It was determined by processing derivative sequences (see Methods) to identify all pixels active above a predetermined threshold during spontaneous events. To enable comparison between strains a uniform threshold was applied to all slices. By this measure, the intensity of EC activation above threshold in LE (57.26% ± 7.99% of pixels) exceeded that in both W (8.27% ± 2.17%) and SD (8.45% ± 2.50%) by a large margin (p < 0.001; one-way ANOVA). The intensity of activation in W and SD slices were not significantly different (Fig 2A).

Lastly we quantified the relative distribution of activity in the lateral and medial regions of EC. It has been proposed that lateral EC and medial EC mediate distinct functions in vivo [26], and it appeared subjectively before the analysis that activity in W and SD slices was often weaker in medial EC. To quantify this, the EC was divided into two equal regions of interest (ROI) and the ratio of “% of lateral activation” to “% of medial activation” was computed. For LE this ratio comes out very close to one (1.34 ± 0.11), consistent with an even distribution of activity throughout both medial and lateral EC. For SD the ratio (10.06 ± 0.83) was significantly higher than LE by a t-test (p<0.001) but not significantly so by one-way ANOVA for all three strains (p>0.05). The lateral-to-medial ratio was significantly greater for W slices (30.17 ± 8.13; p < 0.001; one way ANOVA vs LE), indicating greater lateral compared to medial activation (Fig 2B). The variability in this measure was particularly high in W slices, reflecting the fact that a significant fraction of spontaneous events in W slices involved predominantly medial activation (producing ratio values < 1).

### Comparison of Long Evans and the Brattleboro mutant strain, a model of schizophrenia-like psychopathology

**Fig 3:**
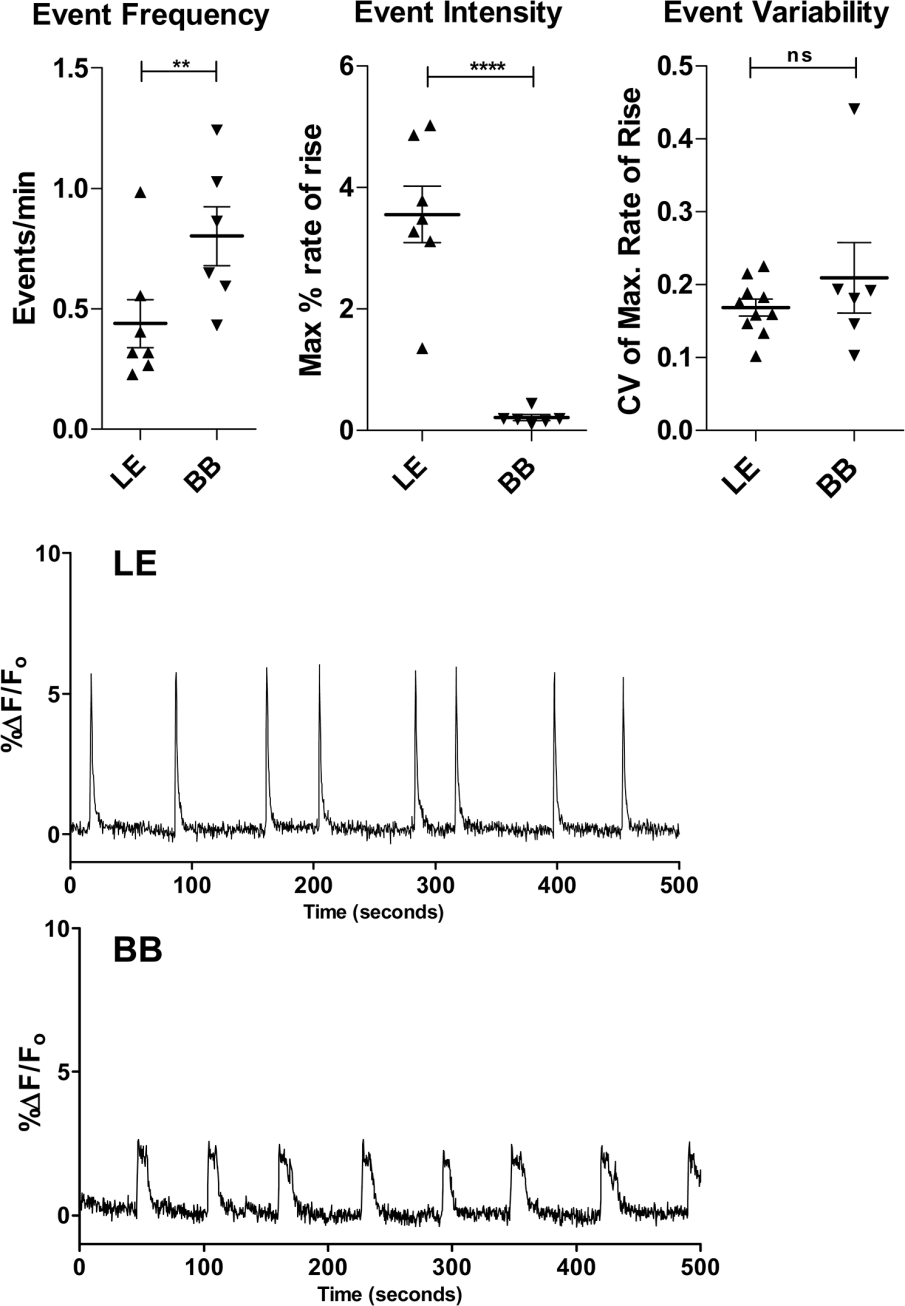
Temporal dynamics of EC spontaneous activity reveals reduced activation in BB relative to LE. A significant increase in the frequency (**A**) of spontaneous events is apparent in BB slices. In contrast, the intensity of activation (**B**) as measured by the maximal rate of change in the calcium signal at the onset of each event is significantly reduced in the low performing strain. The coefficient of variation (CV) in the activation intensity of BB was not significantly increased over that of LE (**C**), demonstrating no increased event-to-event variability in extent of network recruitment. In all cases data are expressed as mean ± SEM. Event variability is based on CVs computed using all events recorded during a calcium imaging sequence. ** p<0.01; ****, p< 0.0001. two-tailed t-test (n’s: LE = 10 slices (7 pups); BB = 6 slices (6 pups)). **D** shows representative traces of the calcium-dependent fluorescence over time taken from active areas of EC in each strain.

In the second part of this study we compared EC activity in slices from the Brattleboro strain (BB) to slices from LE, the control parental strain. BB is a naturally-occurring mutant of the LE strain lacking the neuropeptide arginine-vasopressin (AVP) in the central nervous system. Studies have characterized this strain as a useful model for schizophrenia-like pathology and have demonstrated restoration of normal behavior by the atypical antipsychotic, clozapine [27]. We measured the same parameters described above. Our results show that the EC activation pattern in BB was very significantly different from that of its parent strain (Fig 3). This difference can be summarized as a generalized depression of activity, albeit with a significant increase in event frequency (LE: 0.41 ± 0.05 events/min; BB: 0.80 ± 0.12 events/min; p = 0.04, two-tailed t-test) (Fig 3A). Although higher than LE, this frequency of activation was significantly below that observed in both SD and W.

The intensity of activation in BB slices (0.21 ± 0.05) was very significantly depressed (p< 0.0001; two-tailed t-test) compared to LE (3.55 ± 0.46) (Fig 3B). This level of activation strength was comparable to that recorded in SD (p = 0.34; two-tailed t-test), but was significantly lower than that recorded in W slices (p < 0.0001; two-tailed t-test).

Unlike with W and SD, the variability of activation in BB slices (0.21± 0.05) was not significantly different from that of LE (0.17 ± 0.01; p = 0.18; two-tailed t-test) (Fig 3C).

**Fig 4:**
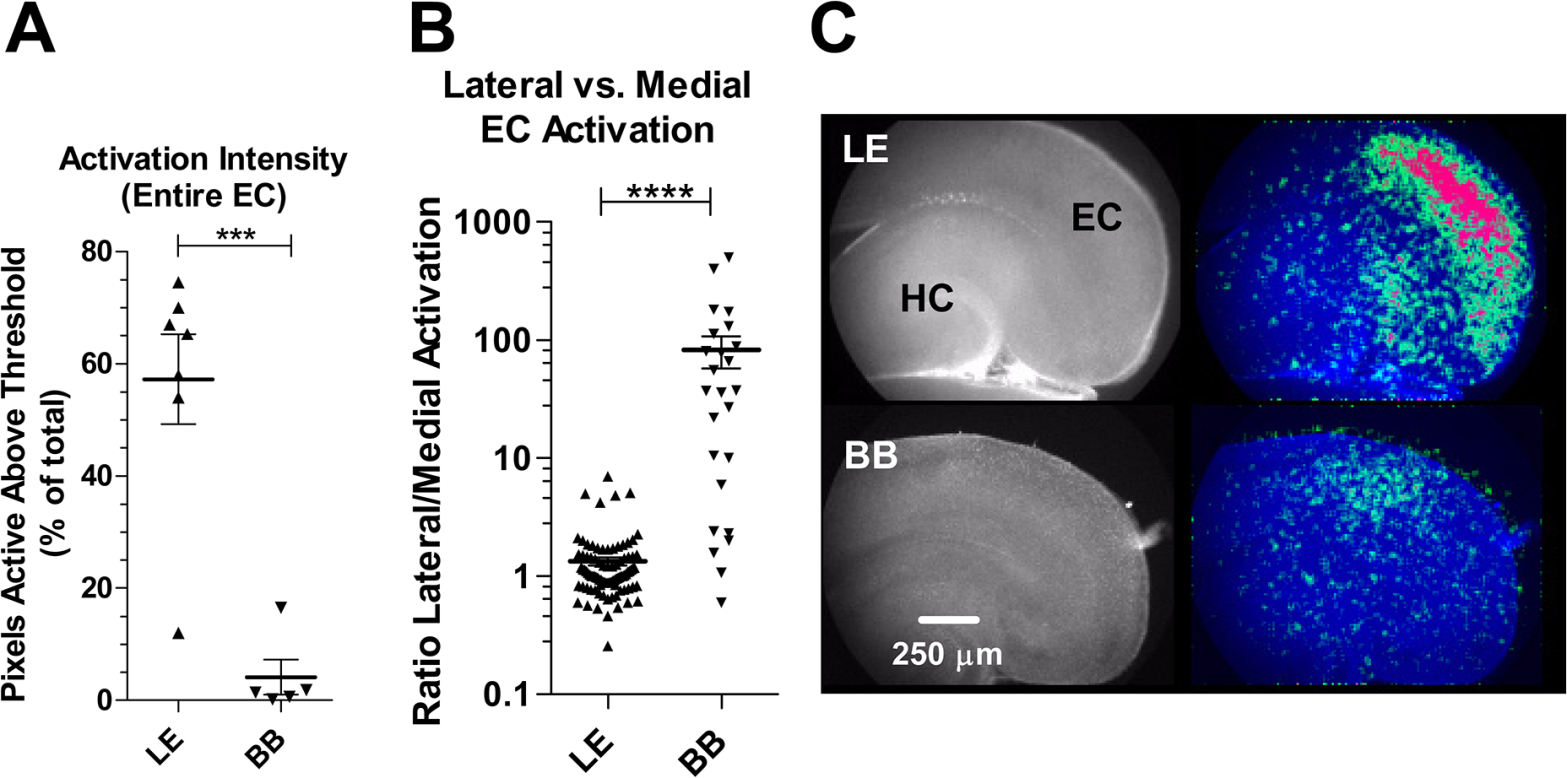
Spatial mapping of EC spontaneous activity reveals severe reduction in BB activation vs. LE. A significant decrease in the overall activation intensity (**A**) of spontaneous events is apparent in BB slices. The intensity of EC activation was calculated as the percentage of total EC pixels active above a threshold value during each coordinated event. **B** quantifies the localization of activity within EC in terms of the relative activation of lateral vs. medial pixels. Activation is uniform throughout EC in LE but biased in favor of lateral activation in BB. *** p<0.001, ****, p< 0.0001. two-tailed t-test (n’s: LE = 10 slices (7 pups); BB = 6 slices (6 pups)). **C** shows representative composite images illustrating the activated pixels (greens and reds in pseudocolor overlay shown on right) in a slice from each strain. HC, hippocampus; EC, entorhinal cortex; In every slice medial is down, posterior is right.

Activation in BB slices registered the most severe evidence of depression relative to LE when assessed in terms of the fraction of pixels active above a threshold level. With a mean activation of 4.14% (± 3.11) this parameter in BB slices registered at a mere 7.2% that of LE slices, where activation averaged 57.26% ± 7.99; (p = 0.0003, two-tailed t-test) (Fig 4A). Even compared to W and SD, which had depressed values for this parameter relative to LE, the BB slices were further depressed by approximately 50%.

Finally, the imbalance in lateral vs. medial activation in BB slices exceeded even the highly asymmetrical values observed in W (p = 0.013; two-tailed t-test; BB vs W). In all cases the imbalance reflected a relative decrease in medial activation. In contrast to the even distribution of activity throughout EC in LE slices (L/M ratio: 1.34 ± 0.11), the L/M ratio in BB slices was 82.3 (± 24.7; p < 0.0001; t-test relative to LE) (Fig 4B). The high s.e.m. in the data for this BB measurement reflects the fact that activation in medial EC was very close to baseline levels, thus increasing the noise in the respective measurement ratios in BB slices.

### A potential role for dopaminergic modulation in generating strain differences

**Fig 5:**
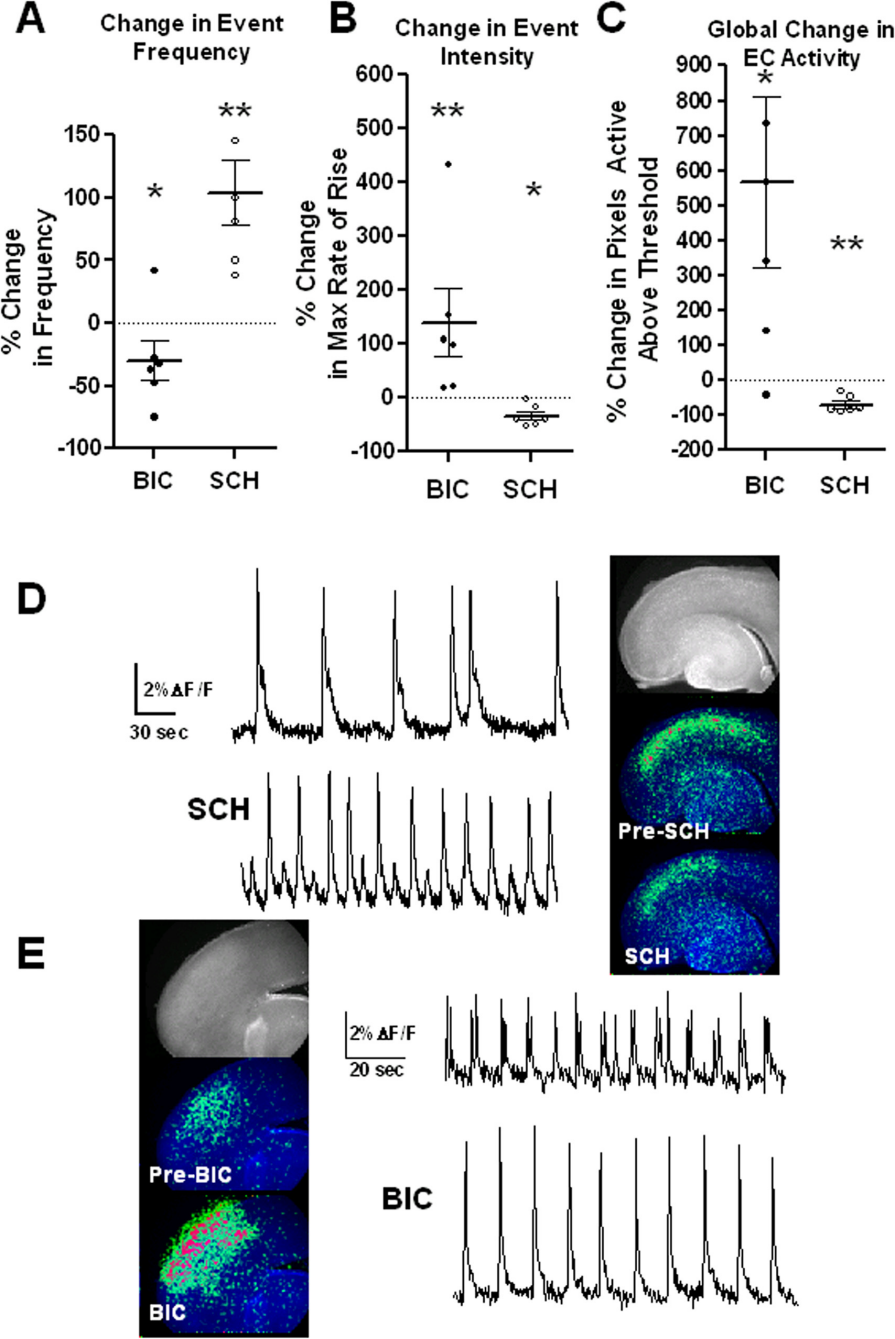
GABA-A and D1/D5 dopamine receptors shape the pattern of EC spontaneous activity. Results of experiments comparing EC activity before and during application of the GABA-A antagonist bicuculline (BIC; 10μM) or the D1/D5 antagonist SCH23390 (SCH; 10μM) show the shifts in the pattern of activation towards or away from an LE-like pattern, respectively. The observed changes in event frequency (**A**), maximal event intensity (**B**), and overall intensity of activation (**C**) in EC are consistent with the view that higher excitation drives activity toward the LE pattern, and higher inhibition drives it toward the pattern found in low performing strains. Representative traces and images of calcium-dependent fluorescence obtained before and during SCH application (**D**) and BIC application (**E**) illustrate the changes observed. BIC experiments: n = 6 slices from 6 SD pups, ages P5-P9. SCH experiments: n = 6 slices from 6 W pups, ages P4-P6. Orientation in all images: lateral is up, caudal is left. * p<0.05; ** p<0.01 paired t-test of mean values obtained before and during antagonist application in each slice.

Our results raise the question of what mechanism(s) may underlie the observed correlation between depressed EC activity early in development and low cognitive performance later in life. Altered cortical GABA-A, glutamatergic and dopaminergic neurotransmission have been implicated in the cognitive dysfunction of schizophrenia [28,29,30], and dopaminergic innervation of EC is reduced in the brains of schizophrenics [31]. Any one of these alterations could result in a disturbed balance between excitation and inhibition in the immature EC. (Note that, unlike in the immature hippocampus, where GABA-A is excitatory, GABA-A inhibits action potential firing and associated calcium transients in the immature EC (see S1 Fig), as in all cortical areas outside hippocampus). Therefore, in the third part of this study we investigated the role of GABA-A receptors and dopamine neuromodulation in shaping the pattern of activity in low performing strains (W and SD) by comparing activity before and during application of antagonists to GABA-A and D1 receptors. We evaluated three of the parameters described above: frequency of events, maximal intensity of activity (local measure), and overall intensity of activity (global measure for all EC). Our results show that activity in the presence of the GABA-A antagonist exhibits significant changes in the three parameters, all of which shifted towards a pattern more typical of the Long Evans pattern (Fig 5). The shift resulting from acute antagonist treatment is only partial, suggesting that other mechanisms are involved and/or that changes in the functional properties of the network have already taken place even at these early ages and cannot be reversed, at least in the short timeframe of these experiments. The results are consistent with the notion that a difference in GABA-A-mediated inhibition could account for differences in the three main parameters of spontaneous activation observed between high and low performing strains.

We then sought to drive the balance in the opposite direction. Increasing inhibition (e.g, with a benzodiazepine agonist; see S1 Fig) or decreasing excitation (e.g. with ionotropic glutamate receptor antagonists) are not useful ways to probe the mechanisms involved in the strain differences described above because both approaches eliminate activity altogether. However, in adult layer II EC neurons D1 receptors have been shown to both reduce gabaergic inhibition [32] and enhance excitatory transmission [33]. Therefore blocking D1-subtype receptors using the D1/D5 antagonist SCH23390 provides a more measured and relevant way to shift the balance in favor of inhibition without eliminating activity entirely. As shown in Fig 5, this manipulation shifts the pattern of activity in EC *away* from the Long-Evans pattern, i.e it depressed intensity and increased frequency. Again, the direction of all changes observed, including the frequency of events, support the view that differences in dopaminergic signaling, through a shift in the excitation/inhibition balance, underlie the observed differences between strains. Our results are therefore consistent with the hypothesis that at early postnatal ages low performing strains exhibit more inhibition/less excitation in EC relative to the high performing strain (LE). Strain differences in dopamine signaling are known to exist in adult rat brain. There is, for example, evidence of altered dopamine signaling in SD relative to LE [34,35]. However, since the relationship between dopamine levels and neural activity (and cognitive performance) is known to follow an “inverted-U-shaped” function [36,37,38] we cannot conclude from our results with SCH23390 that SD, W or BB slices would be expected to have lower dopamine levels than LE, necessarily. Nevertheless, our results suggest a scenario in which these differences in dopamine signaling, if already present early in development, could underlie the differences in the pattern of spontaneous activity among strains.

## Discussion

In this study we examined different rat strains to determine if they express distinct patterns of spontaneous cortical activity during early postnatal life. We deliberately chose strains that are well characterized behaviorally and thus are known to exhibit differences in cognitive performance.

We focused on entorhinal cortex primarily because of its known involvement in many cognitive functions. The widespread connectivity of EC includes reciprocal connections with key cognitive areas such as the hippocampus, prefrontal cortex, and nucleus accumbens. Through these and other projections it is therefore likely that EC plays an important role in a wide range of cognitive processes. Equally important is that this cortical area has a simple, relatively well-characterized pattern of spontaneous network activity early in development. However, since we did not analyze activity in other cortical areas we do not know if these strain-related differences are unique to EC. This is an interesting question that remains to be addressed.

Starting with the initial description of periodic spontaneous electrical discharges in layer 2 neurons of immature EC by Jones and Heinemann [39] other groups have further characterized the coordinated spontaneous spikes and associated calcium transients in the immature EC and have investigated many of the properties and mechanisms involved in mediating this activity [10,40]. We are not aware, however, of any previous characterization regarding strain-related variations in the pattern of these local network events. Therefore the goal of this study was two-fold: to ask if differences in activity patterns between strains can be discerned using relatively low resolution imaging, and if so whether the differences correlate in any straightforward way with known differences in cognitive performance in these strains. In both respects our results are unambiguous and support the view that the pattern of early spontaneous activity, at least in EC, may provide a predictive measure of deficits in cognitive capacity later in life.

While the first part of our study revealed some differences between the two lower performing strains, W and SD, the differences between them were less significant compared to the differences between each of them and the higher performing strain, LE, particularly with respect to the overall strength of EC activation during individual events. The observed differences are consistent with lesser neuronal participation and/or lesser coordination by neurons of the lower performing strains in this early developmental form of activity, suggestive of early alterations in excitability or connectivity, or both. The results of the third part of our study indicate that a simple shift in the balance of excitation and inhibition could account for these differences.

The conclusions derived from the first part of the study are strengthened further by the similar departure from the robust LE activation pattern observed in EC of BB slices obtained in the second part. Activity in BB slices was not only weaker than in LE, but by some measures (e.g. local maximal activation intensity, global activation, the lateral/medial activation ratio) activation in BB slices was weaker than in W and SD slices as well. However, the significance of this further weakness beyond that of W and SD is still unclear. To our knowledge there are no studies comparing side by side the cognitive performance of BB with that of W or SD rats, so we are not able to relate the differences we observed in EC activation to possible differences in cognitive performance between these strains. If weak or poorly coordinated EC activation during development is predictive of impaired performance in adulthood, one might expect that progressively weaker levels of activation will be associated with increasingly severe impairment. We can only speculate as to whether the further weakness observed in BB is related to the reported schizophrenia-like traits of this strain. Nevertheless, it is clear from studies comparing cognitive performance in BB and LE rats that, in accordance with BB being useful as a neuropsychiatric animal model, BB rats perform inferiorly to LE rats in a variety of cognitive tasks [41,42,43,44] and exhibit deficits in prepulse inhibition of the startle reflex [42,44], a measure of sensory gating that may underlie attention deficits and which requires a functioning ventral EC [45,46], the area we focused on. Therefore, the differences we observed between LE and BB, two strains that differ by a single genetic mutation, lend further support to the notion that a predictive relation exists between early network activity and adult cognitive function. While the absence of the excitatory neuropeptide arginine vasopressin in BB brain could potentially explain the altered excitatory/inhibitory balance in this strain we must note that the effect of AVP on developing EC neurons, if any, is not known. On the other hand, the fact that some behavioral deficits in adult BB rats can be reversed with clozapine [27,47], a compound believed to act in part by increasing cortical dopamine release [48], suggests that cortical dopaminergic function may be impaired in this strain.

Looking forward, another question is the potential clinical significance of these findings to human developmental brain disorders such as schizophrenia. Clearly the nature of this type of spontaneous activity, which is intermittent and large-scale (involving the correlated activity of large numbers of nearby neurons), renders it potentially amenable to being recorded with non-invasive brain imaging techniques such as fMRI. Thus, this activity is likely to generate a strong blood oxygen level dependent (BOLD) signal detectable in vivo without particularly high spatial resolution or sampling rates [49]. It is unclear, however, what developmental stage in human EC corresponds to rodent EC during the first postnatal week. Estimates of correspondences across species rely on specific markers and vary somewhat according to the marker used. It is reasonable to expect, however, that an equivalent stage in human EC development occurs prenatally [50,51], in which case it may seem less amenable to functional neuroimaging. However, although challenging, the feasibility of human fetal brain fMRI has been demonstrated and this approach is poised to become a powerful tool to assess prenatal brain activity [52].

To summarize, by comparing different strains, we provide evidence that a potentially useful correlation exists between several simple measures characterizing the pattern of early spontaneous activity in the immature EC in different strains and what is known about the performance of these strains in cognitive tests done in adulthood. As we demonstrate, the mechanistic explanation for these differences during early development may be a simple imbalance in excitation/inhibition caused by different levels of dopaminergic modulation. Replicating this analysis with other rodent strains, including genetic models of human psychopathology, will help obtain a better understanding of the nature and extent of this correlation. Ultimately, since EC as well as other key circuits involved in cognitive and emotional processing are likely to exhibit spontaneous activation patterns during human brain development, these findings imply that characterizing early spontaneous activity in a larger number of local circuits could translate into a useful set of markers for understanding what the early trajectory towards cognitive dysfunction and mental illness looks like in neurophysiological terms. Having the means to detect this trajectory in at-risk individuals, long before crippling forms of cognitive dysfunction take hold, should be a useful aid to understanding and treating these disorders.

## Materials and Methods

All experimental protocols were performed in accordance with the National Institutes of Health Guide for the Care and Use of Laboratory Animals and approved by our Institutional Animal Care and Use Committee.

### Animals

The experiments described here were performed on slices obtained from rat pups between postnatal days 4 and 9. Animals were purchased from Charles River (Wilmington, MA) or Harlan Labs (Indianapolis, IN). Slices from a minimum of 5 animals (range: 5–7) from each strain were used for the analysis of parts 1 and 2. For each strain pups were obtained from two different dams. The total number of spontaneous events analyzed was: 91 for Long Evans, 154 for Sprague Dawley, 142 for Wistar, and 81 for Brattleboro. Total recording time for each strain over all the slices was (in minutes): 261 (LE), 98 (SD), 87 (W) and 97 (BB). All events in any given sequence were used in the analysis provided the activity generated signals exceeding 2 standard deviations of the baseline noise in terms of intensity or maximal rate of rise. In the third part of our study similar numbers of animals were used for each of the two experimental treatments. However, only slices from SD and W animals were employed in these experiments as the intermediate level of activity in these strains provided the best opportunity to observe parameter shifts in either direction.

### Tissue preparation

Slices were cut at 350μm-thickness in ice-cold normal artificial CSF (ACSF) using a vibratome. The composition of the ACSF, modified according to MacGregor et al. [53] to prevent neuronal swelling, was as follows (in mM): 109 NaCl, 2.5 KCl, 1.25 KH_2_PO_4_, 1 MgCl-6H_2_O, 35 NaHCO_3_, 10 glucose, 20 HEPES, and 1 CaCl_2_ (1 mM CaCl_2_, was used since it is a physiological concentration, and one that has been shown to promote the ability of neurons to exhibit sustained firing [54]). The pH after equilibration with 95%_O2_/5%CO_2_ was 7.4. Horizontal slices were obtained at the level of the rhinal sulcus. Only the caudal half of the brain was saved and the two hemispheres were separated to reduce the size of the slice for ease of handling. Because cytoarchitectonic features are not well defined at these young ages, cortical areas were identified primarily by their relative spatial location within the cortex, according to published maps [55,56]

### Fura-2 loading

Loading was performed by bath application of fura-2 acetoxymethyl ester (fura-2 AM; Life Technologies, Grand Island, NY) for 2 h at 30°C in ACSF containing 5 μg/ml fura-2 AM. The indicator dye stock was prepared in DMSO (50 μg in 50 μl) and added to filtered ACSF (0.2 μm). To ensure adequate oxygenation of the submerged slice during dye incubation, the loading chamber (4 cm diameter; 1ml volume of ACSF) was kept in a closed container that was oxygenated continuously with 95% O2/5% CO2 and kept on a rotating platform at 60 rpm.

### Calcium imaging

For imaging, slices were removed from the loading solution and placed in standard oxygenated ACSF, transferred to a submersion recording chamber on the stage of an upright compound microscope (Nikon E600FN), and perfused with oxygenated ACSF at a rate of 4–5 ml/min in a temperature-controlled (29±1°C) perfusion chamber (volume, 200–400 μl; Warner Instruments, Hamden, CT). Slices were viewed with a 3.5x air objective. Epifluorescence imaging of fura-2 intensity was performed with a 75W Xenon light source and a low-light CCD digital camera. (1024x1280 pixel, 12.5 MHz, 12-bit SVGA Sensicam; Cooke Corp., Tonawanda, NY). A 0.5x adapter was placed in the camera port to capture a larger field (up to 3 x 2.4 mm) on the CCD sensor. Minimal exposure of the preparation to the excitation light was achieved by controlling a shutter in the excitation light path (Ludl Electronics, Hawthorne, NY), reducing light intensity with neutral density filters, and operating the camera on a 8x8 pixel-binning mode to increase sensitivity. All measurements of relative changes in [Ca^2+^]i were made at a single excitation wavelength using a 380±5 nm bandpass filter (Chroma Technology, Brattleboro, VT). Emission fluorescence was filtered with a 480±20 nm bandpass filter. Acquisition protocols consisted of 10- or 20-min-long time-lapse sequences (250 ms integration times per frame; 0 ms interval) of fura-2 fluorescence. Custom scripts were written in IPLab software (Scanalytics, Fairfax, VA) running on Windows XP. Because of the longer recording times typically needed for the bicuculline and SCH23390 experiments lower illumination intensity, longer exposures and a 400nm longpass emission filter were used to minimize possible photodamage.

### Drug application

SCH23390 (10 μM) and Bicuculline (10μM) were applied by a multi-valve, single-output gravity perfusion system (ALA Scientific, Westbury, NY). Twenty to 30 minutes of baseline activity were recorded prior to application of the antagonist. Five minutes of perfusion with antagonist were allowed prior to recording its effects for an additional 10–30 minutes.

### Analysis of calcium imaging data

As mentioned above, measurements of changes in fura-2 fluorescence ([Ca^2+^]i) as a function of time were done at a single wavelength, not by the wavelength ratio method, to enable faster sampling rates. All analysis and processing, as well as playback of the image sequences for visual inspection, was made using custom scripts written in IPLab software. Regions of interest (ROI; 200 μm x 200 μm) over EC were selected, and the mean pixel intensity at each frame was measured. Raw data were in the form of a linear 12-bit intensity scale (0–4096). These data were first plotted as fluorescence intensity versus time and subsequently converted, for comparison across experiments, to a relative scale (%∆*F*/*F*_baseline_). The script written for conversion to a relative scale also de-trended the baseline and inverted the polarity of the signal, thus converting negative changes in fura-2 fluorescence (reflecting increases in [Ca^2+^]i) to positive changes in %∆*F*/*F*. To visualize the spatial changes in calcium resulting from spontaneous activity, the raw sequences were processed to highlight changes in fluorescence intensity between frames. Sequences representing the first derivative of the fura-2 fluorescence were obtained by processing an entire raw sequence of images with an algorithm that subtracted the value of each pixel in each frame from the value of that pixel on the previous frame. In this new sequence, pixel intensities represent the magnitude of *changes* in 380 nm fluorescence; pixels that changed (decreased) the most had higher values in the processed images. Because at low magnification the change in [Ca^2+^]_i_ signal reflects the magnitude of the calcium change in individual cells as well as the number of cells participating in a spontaneous event, the magnitude of the average rate of increase in pixel intensity within a ROI at the onset of each event provides a useful measure of activation intensity at that location. To obtain a measure of the intensity of activation over the entire EC an algorithm was used that counted the number of pixels whose change in intensity during each event exceeded a threshold value. The number of pixels was then converted to a percentage of the total number of pixels in the area comprising the entorhinal cortex (EC). To allow comparison across slices and across strains the same threshold value was used for all slices. The EC outline was drawn manually for each sequence prior to running this algorithm and included all EC layers spanning the latero-medial extent, from the slight constriction corresponding to the rhinal sulcus to the most medial end of EC. This area was divided into two parts at the midpoint when computing the lateral-to-medial ratio of activation. In cases where EC activation occurred over more than one frame (due to the fact that spontaneous activity propagates through EC at variable speeds) activated pixels from all frames having an increase in activity where included in the calculation.

### Statistical Analysis

Comparisons were made with two-tailed unpaired t-test, one way ANOVA with Bonferroni post test for multiple comparisons or paired t-test as indicated. For statistical tests based on individual events the data were alternately grouped as means of all events from each animal of a strain and as individual data points obtained from all animals from a strain. Of these two ways of grouping the data we report the result that yielded the lower significance level. For coefficient of variation analysis only recording sequences having 4 or more events were used. All tests were performed using the statistical functions of GraphPad Prism 5 for Windows software package version 5.04. All plots show the mean ± SEM

## Supporting Information

S1 Fig Inhibitory effect of diazepam (DZP; 200 nM) on EC spontaneous activity shows that GABA-A is not excitatory at this age. Traces show spontaneous calcium transients recorded in EC before, during and after (wash) bath application of the non-selective positive allosteric modulator of GABA-A receptors, diazepam, in a P5 Wistar slice.
(TIF)
