# Supplementary figures and images for: Patterns of Spontaneous Local Network Activity in Developing Cerebral Cortex: Relationship to Adult Cognitive Function
Source: PLoS One. 2015 Jun 22;10(6):e0131259. doi: 10.1371/journal.pone.0131259 (PMC4476761; doi:10.1371/journal.pone.0131259)

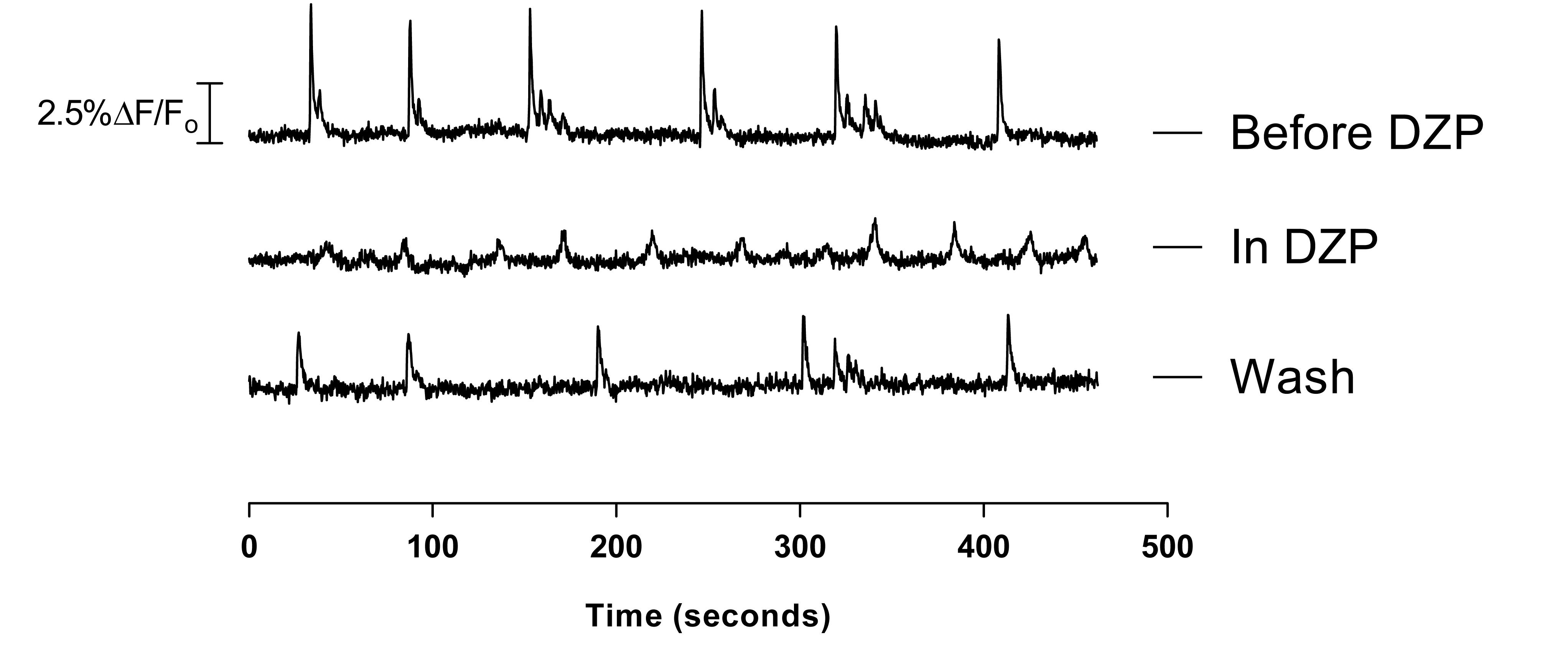

Supplement: S1 Fig — Traces show spontaneous calcium transients recorded in EC before, during and after (wash) bath application of the non-selective positive allosteric modulator of GABA-A receptors, diazepam, in a P5 Wistar slice. (TIF) [file pone.0131259.s001.tif]
